# Strategies for intraoperative management of the trigeminal nerve and long-term follow-up outcomes in patients with trigeminal neuralgia secondary to an intracranial epidermoid cyst

**DOI:** 10.3389/fsurg.2022.930261

**Published:** 2022-07-28

**Authors:** Zhenyu Zhang, Wenhua Wang, Feng Yu, Sze Chai Kwok, Yuhai Wang, Jia Yin

**Affiliations:** ^1^ Department of Neurosurgery, Shanghai Tenth People’s Hospital, Tongji University School of Medicine, Shanghai China; ^2^ Department of Neurosurgery, 960 Hospital of The People's Liberation Army of China, Jinan, China; ^3^ Shanghai Key Laboratory of Brain Functional Genomics, Key Laboratory of Brain Functional Genomics Ministry of Education, Shanghai Key Laboratory of Magnetic Resonance, Affiliated Mental Health Center (ECNU), School of Psychology and Cognitive Science, East China Normal University, Shanghai, China; ^4^ Division of Natural and Applied Sciences, Duke Kunshan University, Kunshan, China; ^5^ Shanghai Changning Mental Health Center, Shanghai, China; ^6^ Department of Neurosurgery, 904 Hospital of The People's Liberation Army of China, Wuxi, China

**Keywords:** Cerebellopontine angle, epidermoid cyst, trigeminal neuralgia, management strategy, trigeminal nerve

## Abstract

**Background:**

Epidermoid cysts (ECs) are one of the most common causes of secondary trigeminal neuralgia (TGN). However, most previous studies have primarily focused on whether complete tumor resection was achieved, and few studies have discussed the primary goal of pain relief.

**Objective:**

The present study provides intraoperative strategies for trigeminal nerve (TN) management in patients with TGN secondary to an EC and observed long-term follow-up outcomes.

**Methods:**

A total of 69 patients with TGN secondary to an EC at our hospitals were included (January 2011–June 2021). The same surgical team performed all surgeries using a retrosigmoid approach. After EC removal, different methods for TN management were used, including microvascular decompression (MVD), sharp capsulectomy, nerve combing and embedded cholesterol crystal excision. The epidemiological, clinical, and surgical data were extracted.

**Results:**

The total EC removal rate was 92.8% (64/69). All patients achieved initial pain relief postoperatively, and 12 patients (17.4%) experienced varying degrees of hemifacial hypesthesia, which was relieved within 3–6 months. Three patients (4.3%) reported partial pain recurrence within a median follow-up period of 5.5 (0.5–10.5) years, which was relieved completely after low-dose carbamazepine administration.

**Conclusion:**

The primary goal of surgical tumor removal for patients with TGN secondary to an EC is relief of the main symptom of tormenting pain. The selection of an appropriate strategy for TN, including MVD, sharp capsulectomy, nerve combing or embedded cholesterol crystal excision, should depend on the patient's situation.

## Introduction

The incidence of intracranial epidermal cysts (ECs), also named intracranial cholesteatomas, accounts for approximately 1% of all intracranial tumors (1–3), and these tumors primarily occur in the cerebellopontine angle (CPA) (4, 5). EC is one of the main causes of secondary trigeminal neuralgia (TGN) (6, 7). The curative goal of patients with TGN secondary to an EC is tumor removal with recurrence prevention and avoidance of complications (8–10). However, the primary goal of treatment in patients with TGN as the first or main symptom is relief of the tormenting pain (11, 12). When pain is not relieved, even complete resection of the tumor should be considered a treatment failure, at least from the patient's perspective. Therefore, the relief of pain, reduction of facial numbness and other symptoms of cranial nerve (CN) injury, and prevention of TGN recurrence using appropriate approaches for trigeminal nerve (TN) management during the process of tumor resection are more important than tumor resection. Few studies have summarized and discussed experience and practice in this respect. This article summarizes our 10-year experience and practice in the treatment of 69 patients with TGN secondary to an EC and discusses the preferred management strategies.

## Methods

### Patient characteristics

**Figure 1:**
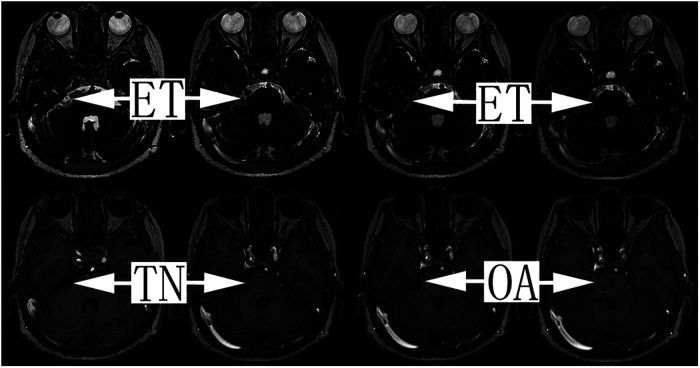
The 3D-FIESTA and 3D-TOF magnetic resonance sequence of TGN secondary to EC patients with offending arteries. Tumors of the epidermoid cyst, trigeminal nerve and offending artery are indicated by the arrows. ET, epidermoid tumor; TN, trigeminal nerve; OA, offending artery.

All patients who underwent surgery for TGN at our hospitals from January 1, 2011, to June 30, 2021, were reviewed retrospectively. The diagnosis of TGN was made in 2,048 cases according to the criteria for classic TGN (13.1.1) of the International Classification of Headache Disorders 3 (ICHD-3). As part of the routine clinical management of TGN, all patients underwent a preoperative three-dimensional time-of-flight (3D-TOF) magnetic resonance imaging (MRI) examination. In cases of prepontine cistern enlargement or abnormal signals, contrast enhancement or 3D fast imaging using steady-state acquisition (3D-FIESTA) was applied (Figure 1) (13). Of the 2,048 consecutive TGN cases, the EC was diagnosed secondary to TGN in 76 cases preoperatively, surgically and pathologically. By the end of June 2021, seven patients were lost to follow-up, and 69 were ultimately included in this study for analysis. Seventeen of the 69 patients had undergone other surgical treatments (percutaneous radiofrequency thermocoagulation, percutaneous ball compression or *γ*-knife surgery) before admission to our hospitals, and nine of these patients had obvious postblock hypesthesia. The ethics committee of the two hospitals involved approved this study. The patient data were anonymous, and informed consent was not necessary.

### Anatomical observations and surgical procedures

All patients underwent treatment with a standard suboccipital retrosigmoid approach. After the release of cerebrospinal fluid (CSF) under a microscope and retraction of the cerebellar hemisphere, a pearl-like space-occupying lesion with an intact capsule was observed at the CPA. The TN, facial-acoustic nerve, and lower CNs were compressed and encapsulated. After opening the arachnoid membrane, intracapsular resection was performed as the primary procedure, including clearing of the cholesteatomatous tumor tissue in all corners using an aspirator and dissector and the tumor tissue on the tentorium cerebellum and in the contralateral prepontine cistern by adjusting the angle of the microscope. For tumor capsules attached to the brain stem, compulsory resection was not performed to avoid damaging the penetrating vessel, which results in severe consequences. In cases where the tumor wrapped the facial-acoustic, abduction and trochlear nerves, a small aspirator tube with a well-controlled suction force was used because these nerves are thin and delicate, and excessive suction could cause direct damage. The adhered tumor capsule was cut and removed *via* sharp dissection. After clear and complete tumor removal, primary attention was focused on the management of the TN root. Different surgical procedures for TN management were performed according to the type classification, as discussed below.

### Type I

**Figure 2:**
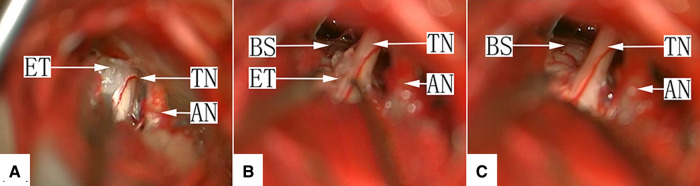
(**A**) Type Ia: After craniotomy and retraction of the cerebellar hemisphere under a surgical microscope, the facial-acoustic nerve, trigeminal nerve and epidermoid cyst in the lateral aspect of the prepontine cistern are seen. The tumor tissue appears white with a pearl-like luster. TN, trigeminal nerve; ET, epidermoid tumor; AN, acoustic nerve. (**B**): Type Ia: Most cholesteatoid tumor tissue in the prepontine cistern has been removed, with a small amount of tumor residue on the dorsal aspect of the TN root entry zone. TN, trigeminal nerve; ET, epidermoid tumor; AN, acoustic nerve; BS, brain stem. (**C**): Type Ia: The tumor tissue was removed completely, and the TN surface is smooth. TN, trigeminal nerve; AN, acoustic nerve; BS, brain stem.

There were 49 cases of this type, consisting of only an EC without vascular involvement of the TN. Eighteen cases were type Ia, in which the main portion of the tumor was located in the prepontine cistern, and the TN was pushed from the inside to the outside and became distorted but was not completely encapsuled by the tumor. When the arachnoid membrane was opened, the TN root was directly observed, and the running direction of the fiber was seen clearly. The first step in these cases was clearing the tumor portion in the CPA. TN management started by examining and clearing the root entrance/exit zone and the portion entering the Meckel cave, especially the ventral side of the nerve, to avoid missing the tumor outside of the direct surgical microscopic field. The tumor capsule attached to the TN was removed *via* sharp dissection using microscopic scissors (Figure 2, Supplementary Video S1).

**Figure 3:**
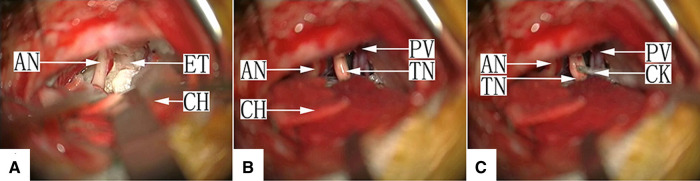
(A) Type Ib: After craniotomy and retraction of the cerebellar hemisphere under a surgical microscope, the facial-acoustic nerve is seen, the depth of which is filled with pearl-like tumor tissue, with the petrosal vein and TN buried inside. ET, epidermoid tumor; AN, acoustic nerve; CH, cerebellar hemisphere. (**B**) Type Ib: The tumor in the cerebellopontine angle (CPA) was removed completely. The TN in the cistern segment is cylindrically round, and its surface is covered with a pseudo-membrane. The longitudinal nerve fibers are not visible. TN, trigeminal nerve; PV, petrosal vein; AN, acoustic nerve; CH, cerebellar hemisphere. (**C**) Type Ib: Nerve combing of the TN. The cistern segment is performed by inserting the combing knife into the nerve and moving it longitudinally. TN, trigeminal nerve; AN, acoustic nerve; CH, cerebellar hemisphere; CK, combing knife.

Twenty-four cases were type Ib, in which the tumor tissue completely encapsulated the nerve. After retraction of the cerebellum, only the tumor was visualized directly in most cases, but the facial-acoustic nerve was seen in some cases. After partial clearing of the tumor, the normally flat TN cisternal segment had become cylindrically round like an electrical pole, with a layer of capsule-like tissue on the surface. The longitudinal nerve fibers could not be clearly identified. Simple tumor resection in cases of severe TN root invasion by the tumor may not relieve pain completely. The nerve combing procedure was applied to release the TN cisternal segment in 11 patients by making 4–8 incisions along the longitudinal axis using a homemade combing knife (Figure 3, Supplementary Video S2).

**Figure 4:**
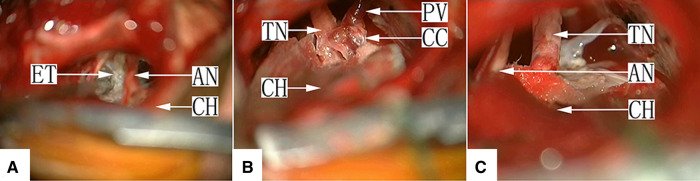
(**A**) Type Ic: After craniotomy and retraction of the cerebellar hemisphere under a surgical microscope, the facial-acoustic nerve is seen, the depth of which is filled with the pearl-like tumor tissue, and the TN is buried inside. AN, acoustic nerve; CH, cerebellar hemisphere; ET, epidermoid tumor. (**B**) Type Ic: The tumor tissue in the CPA was excised, and the TN root closely adhered to a cholesteatoid crystal with a petrosal vein on the surface. CH, cerebellar hemisphere; PV, petrosal vein; TN, trigeminal nerve; CC, cholesteatoid crystal. (**C**) Type Ic: The trigeminal nerve was decompressed completely after removal of the cholesteatoid crystal embedded at the TN root *via* sharp dissection at the expense of the petrosal vein. CH, cerebellar hemisphere; AN, acoustic nerve; TN, trigeminal nerve.

Seven cases were type Ic. Because there may be cholesterol crystals in this type of EC tumor, these stone-like hard substances may be embedded in the TN root, and sharp dissection with microscopic scissors is required to achieve sufficient decompression (Figure 4, Supplementary Video S3).

### Type II

**Figure 5:**
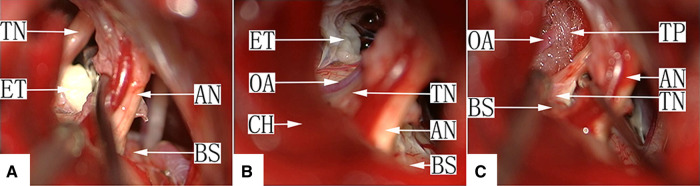
(**A**) Type IIa: After craniotomy and retraction of the cerebellar hemisphere under a surgical microscope, the facial-acoustic nerve and the epidermoid cyst in the prepontine cistern in the median aspect of the nerve are seen. After removal of the tumor tissue, TN was observed. AN, acoustic nerve; TN, trigeminal nerve; ET, epidermoid tumor; BS, brain stem. (**B**) Type IIa: After removal of most of the epidermoid cyst in the prepontine cistern, the TN root was exposed. Two arteries are seen on its ventral side, which created compression on the nerve. AN, acoustic nerve; TN, trigeminal nerve; ET, epidermoid tumor; BS, brain stem; OA, offending artery; CH, cerebellar hemisphere. (**C)** Type IIa: Microvascular decompression was performed by separating the offending artery (OA) at the TN root using a Teflon pad. AN: acoustic nerve; TN: trigeminal nerve; BS: brain stem; OA: offending artery; TP: Teflon pad.

**Figure 6:**
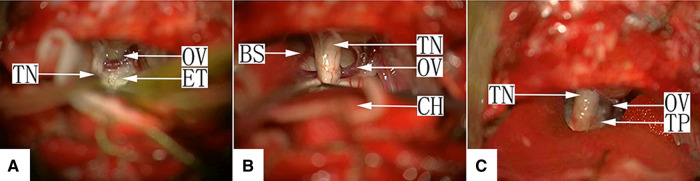
(**A**) Type IIb: After craniotomy and retraction of the cerebellar hemisphere under a surgical microscope, the facial-acoustic nerve, TN and the epidermoid cyst in the prepontine cistern in the median aspect of the nerve are seen. The tumor tissue appears white with a pearl-like luster, and a petrosal vein branch is seen on the surface. TN, trigeminal nerve; ET, epidermoid tumor; OV, offending vein; (**B**) Type IIb: The tumor tissue in the CPA was removed, and a petrosal vein branch is seen intraneurally passing through the TN root. TN, trigeminal nerve; OV, offending vein; BS, brain stem; CH, cerebellar hemisphere. (**C**) Type IIb: Microvascular decompression was performed by separating the petrosal vein branch intraneurally passing through the TN root using a Teflon pad on both sides. TN, trigeminal nerve; OV, offending vein; TP, Teflon pad.

There were 20 cases of this type, in which the TN root contacted the blood vessel during EC excision. Thirteen of these cases were type IIa, in which an offending artery (OA) was found, and microvascular decompression (MVD) was performed (Figure 5, Supplementary Video S4). The remaining 7 cases were type IIb, in which an offending vein (OV) was found. OVs smaller than 1 mm were usually snipped after electrocoagulation, and MVD was used for relatively thick OVs (Figure 6, Supplementary Video S5).

Follow-up evaluations were performed 6 and 12 months post-operatively and then annually thereafter and included routine physical examination, MRI and facial sensory testing in most cases. Telephone interviews were performed if the patients failed to visit our clinic at the scheduled time. In addition to other CN complications, we primarily evaluated the initial pain relief, pain recurrence, and any type of sensory disturbance using the Barrow Neurological Institute (BNI) pain intensity score and the facial numbness score (14). Complete pain relief was defined as a BNI pain score of I. Partial pain relief was defined as a BNI pain score of II or III.

## Results

Sixty-nine of the 2,048 cases were included in the study. The mean age of the 69 EC patients was 49.8 (17–77) years. Most patients were women (68%, 47/69). The mean disease duration was 61.5 (1–480) months, and the mean follow-up duration was 5.5 (0.5–10.5) years. The affected areas and other clinical characteristics are shown in Table 1.

**Table 1:** Clinical characteristics of the patients.

	Cohort size (*n* = 69)
Affected side, No. (%)	
Right	27 (39.1%)
Left	42 (60.9%)
Symptom	
Cranial nerve V	
TGN territory involved	
V1	6
V2	9
V3	13
V1 + 2	5
V2 + 3	26
V1 + 2 + 3	10
Post-block hypesthesia	9
Cranial nerve VII	
Hemifacial spasm	3
Cranial nerve VIII	
Hearing difficulty	5
Tinnitus	3

**Figure 7:**
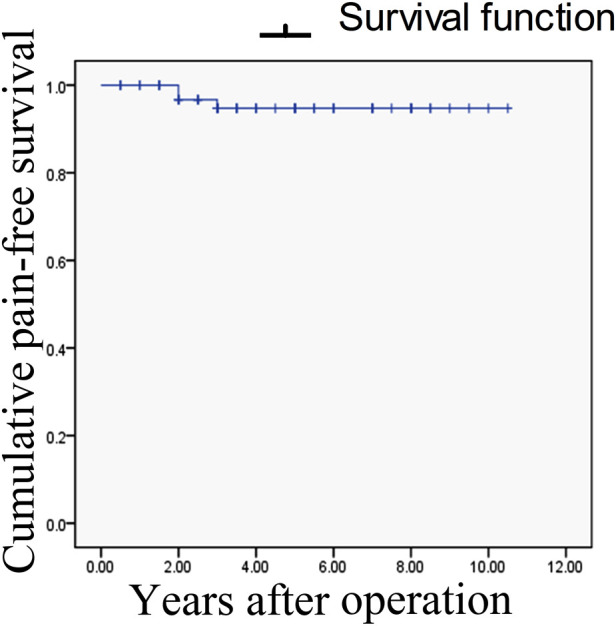
Kaplan–Meier survival pain-free analysis. Recurrence of pain occurred in three patients (at 2, 2, and 3 years post-operatively).

All patients had sutures removed and were discharged 5–7 days post-operatively. Immediate and complete pain relief (BNI pain score I) was achieved in all 69 cases. Postoperative MRI demonstrated total resection in 64 cases and subtotal resection in the other five cases because the tumors on the supratentorial and contralateral CPA parts were relatively large. The main complication was unilateral facial hypesthesia, which occurred as a new symptom or exacerbation of the original bradyesthesia in 12 cases, including three of the 11 type Ib EC patients who underwent combing. This bradyesthesia was relieved 3–6 months post-operatively in all patients. Other presenting complications included acute subdural hematoma in three cases (detected on routine CT checkup 24 h post-operatively), which presented as a thin-layer hematoma with no clinical symptoms and were absorbed spontaneously within one month in all cases, aseptic meningitis in eight cases, diplopia in seven cases, including trochlear nerve injury in four cases and abducens nerve injury in three cases, and hearing loss in five cases. During the follow-up period of 0.5–10.5 years, no significant enlargement of the tumor was observed in the five patients in whom subtotal resection was achieved. Three patients (4.35%) reported partial pain recurrence (BNI pain score II–III) two and three years post-operatively. Treatment for recurrence in these patients included the use of low-dose carbamazepine (<200 mg/d), which provided a satisfactory analgesic effect. Pain-free survival was defined as the time to recurrence of facial pain after surgery. Kaplan–Meier curves were constructed based on pain-free survival (Figure 7). The different types of TN involvement and the occurrence of related complications are shown in Table 2.

**Table 2:** Different types of TN involvement and the occurrence of related CN complications and surgical outcomes.

	Subtotal (*n* = 5)	CN complications (*n* = 20^a^)			
		IV (*n* = 4)	V (*n* = 12)	VI (*n* = 3)	VII (*n* = 5)
Type I					
a (*n* = 18)	1	1	4	1	2
b (*n* = 24)	3	2	5		2
c (*n* = 7)			2		
Type II					
a (*n* = 13)	1	1	1	2	
b (*n* = 7)					1

^a^ As some patients had injury to multiple cranial nerves after operation, the sum of the cranial nerve cases (*n* = 24) is larger than the total number of the complication cases (*n* = 20).

## Discussion

The occurrence of ECs in the CPA is rare, but we have treated numerous cases at our TGN center because the most common onset symptom is TGN. The 69 patients with TGN secondary to an EC included in our series were primarily characterized by a young mean age, pain in the V2 and V3 regions, and a similar onset of symptoms and typical vascular compression presenting as simple TGN (15). The total tumor removal rate was 92.8% (64/69). The simple lateral suboccipital retrosigmoid approach may not expose the distal part of the tumor, and the combined use of the anterior transpetrosal approach or combined transpetrosal approach may be necessary (16, 17); alternatively, endoscopy may be a better means of completely removing the tumor (18, 19). However, a combined approach in this area may cause unexpectedly extensive trauma and require more surgical time. The incidences of perioperative complications, sequalae and tumor recurrence were low in our series. Other than the symptoms of TGN, the symptoms of injury to other CNs, such as diplopia, were restored within approximately six months in most cases. While restoration of hearing loss is relatively difficult, unilateral hearing loss does not have a significant impact on daily activities.

The key for the successful treatment of TGN secondary to an EC is pain relief. The immediate pain relief rate was 100% in our study, and long-term follow-up observation only revealed partial pain recurrence in three cases (4.3%). The incidence of nerve-related complications was also low. Hemifacial hypesthesia was the main symptom, but its postoperative clinical presentation was mild and caused an insignificant impact on patients' daily activities. The symptoms were relieved in 3–6 months in most patients.

A few studies have reported research on TGN secondary to an EC. Kabata (2002) analyzed 28 cases and emphasized that straightening the neuraxis by cutting and removing the adherent tumor capsule and arachnoid membrane was mandatory for the relief of pain and prevention of pain recurrence. With a mean follow-up period of 11.5 years, they detected pain recurrence symptoms in three patients (10.7%). However, their postoperative TN injury rate was as high as 64.3% (18/28). Together with other CN (III-V) injuries, the overall incidence accounted for approximately two-thirds of all patients. Therefore, an excellent outcome was achieved in only 57.1% (16/28) (20). Guo (2011) reported 48 similar cases in which TGN was not relieved after surgery in two cases (a second surgery was performed), and pain recurred in another patient. They noted that resecting the attachment of the tumor capsule to CN V was critical for relieving pain, although this method may damage the nerve (21). Other studies on ECs have only reported intracranial resection of the tumors, with few cases of TGN as the onset symptom (22, 23). These studies demonstrate that the pain relief rate in the management of TGN secondary to an EC is not sufficient, and the rate of TN injury remains high. The etiology of TGN secondary to an EC must be identified and eliminated to achieve the goal of pain relief.

The pathogenesis of TGN secondary to an EC is likely related to the following three conditions: (1) nerve compression from vascular compression; (2) direct compression of the nerve by the tumor; and (3) irritation of the chemicals within the EC or aseptic inflammation of the TN. These factors may work independently or synergistically on the TN to cause demyelination of the nerve and formation of pseudosynaptic connections, which ultimately result in abnormal TN tactile conduction (23, 24).

The principle of our surgical strategy for TN management is removal of the above factors.

1. Strategy for TGN management. Some researchers have advocated conservative management strategies for ECs involving the CN (25). We partially agree that caution should be taken in handling CNs other than the TN, such as the trochlear, abduction, facial and auditory nerves, and use of an aspirator should be avoided. However, pain relief is the primary goal, and we advocate a more positive attitude in treating the TN using a proper method. The present study primarily describes three strategies for TGN management in different situations: (1) sharp dissection of the tumor capsule adhering to the nerve; (2) excision of embedded cholesterol crystals; and (3) combing of the nerve wrapped by the pseudocapsule inside the tumor. Lagares reported that TNs within ECs may undergo pathological changes, including axonal loss and demyelination, with the presence of abundant collagen infiltrates and myelin debris. Myelin-denuded axons have been found in close apposition in some areas, allowing axon-to-axon interactions (26). These findings are consistent with those of previous studies on patients with vascular compression-related TGN. Therefore, nerve combing is generally used instead of MVD when no OV is found (27–29). We used nerve combing to destroy the pseudomembrane formed after nerve demyelination and release the pseudomembrane wrapping the nerve to ensure pain relief.
2. OVs may cause TGN in EC patients. The only controversy is that some researchers believe that the OV is the only cause (30). We found that 30% (20/69) of the cases in our series had OVs, which we carefully examined. An attentive review of the TN by 3D-TOF MRI may be helpful, especially near the TN root entry/exit zone and Meckel cave. With respect to OV management, an OV smaller than 1 mm in diameter may be sacrificed by cutting after cauterization, but removal should be avoided for relatively large OVs (>2 mm in diameter) to reduce hemorrhage and the risk of infarction, which is different from the opinion advocated in most studies that all TGN-related OVs should be sacrificed.

The present study has some limitations. First, it was only a retrospective study. Second, TN combing is an innovative method that has not been generally accepted. Therefore, more studies are required to provide verification of its efficacy and long-term outcomes.

## Conclusion

The primary goal of EC removal as a treatment is relief of the tormenting pain. Caution should be taken to avoid oversight of any possible OVs for decompression. The selection of an appropriate strategy for TGN, including sharp capsulectomy, nerve combing or embedded cholesterol crystal excision, should depend on the extent of tumor invasion into the TN.

## Data Availability

The original contributions presented in the study are included in the article/**Suplementary Material**, further inquiries can be directed to the corresponding author/s.
